# A bibliometric analysis of the Fasting-Mimicking Diet

**DOI:** 10.3389/fnut.2024.1328450

**Published:** 2024-01-23

**Authors:** Xiaoxiao Lin, Yue Gao

**Affiliations:** ^1^Department of Geriatrics, Affiliated Hangzhou First People’s Hospital, School of Medicine, Westlake University, Hangzhou, Zhejiang, China; ^2^Zhejiang Key Laboratory of Traditional Chinese Medicine for the Prevention and Treatment of Senile Chronic Diseases, Hangzhou, Zhejiang, China

**Keywords:** Fasting-Mimicking Diet (FMD), bibliometric analysis, research h-otspots, human health, adaptation

## Abstract

The Fasting-Mimicking Diet (FMD) is a nutritional strategy that involves significantly reducing calorie intake for a specific period to mimic the physiological effects of fasting while still providing the body with nutrition. Our study aimed to conduct a bibliometric study to explore the latest publishing trends and areas of intense activity within the sphere of FMD. We extracted data on FMD publications from the Web of Science Core Collection (WOSCC) database. The bibliometric analysis was conducted by WOSCC Online Analysis Platform and VOSviewer 1.6.16. In total, there were 169 publications by 945 authors from 342 organizations and 25 countries/regions, and published in 111 journals. The most productive country, organization, author, and journal were the United States, the University of Southern California, Valter D. Longo, and Nutrients, respectively. The first high-cited document was published in Ageing Research Reviews and authored by Mattson et al. In this study, they discuss the various health benefits of FMD including improved metabolic health, weight management, and even potential effects on delaying aging processes and reducing the risk of chronic diseases. In conclusion, our study is the first bibliometric analysis of the FMD. The main research hotspots and frontiers were FMD for cancer, FMD for metabolic-related diseases, and FMD for cognitive improvement. FMD may have some potential benefits for multiple diseases which should be further investigated.

## 1 Introduction

The Fasting-Mimicking Diet (FMD) is a dietary regimen designed to replicate the effects of traditional fasting while still allowing for food intake. This approach typically involves a significant reduction in calorie intake, with a specific focus on a low-protein, low-carbohydrate, and high-fat composition, rich in nutrients. FMDs are generally followed for short periods, typically ranging from 3 to 7 days, and are often repeated cyclically, such as monthly, to achieve ongoing benefits. The primary goals of an FMD are to trigger beneficial cellular and metabolic responses similar to those obtained through water-only fasting, such as improved metabolic health, reduced inflammation, and potential longevity benefits. FMD is recommended to be undertaken with medical supervision, particularly for individuals with existing health conditions or those on medications (1–16).

In recent years, FMD become more and more popular since it may have potential benefits for human health, including cancer and diabetes. However, there lacks a bibliometric analysis that consolidates existing publication patterns and forecasts emerging research focal points in this domain. Bibliometric Analysis refers to the quantitative assessment and interpretation of scientific literature (17–19). It uses statistical methods to analyze articles, books, and other publications to provide insights into the patterns, structures, and trends in scientific research. Essentially, bibliometrics play a crucial role in deciphering the influence, distribution, and development of research across diverse fields. By employing bibliometric Analysis, one can gain clarity on pivotal articles, the trajectory of research trends, and the progressive unfolding of scientific concepts throughout history. In light of this, our study aimed to undertake an exhaustive bibliometric analysis to identify the leading edges and areas of concentrated research activity in the FMD field.

## 2 Methods

### 2.1 Data collection

Relevant literature pertaining to the FMD was systematically sourced from the Web of Science Core Collection (WoSCC). Recognized for its comprehensiveness and authority, WoSCC served as the primary database for this study. The search was delimited to documents published between 1 January 2000 and 15 September 2023. The specific search terms employed for this literature hunt were: “Fasting-Mimicking Diet” OR “Fasting-Mimicking Diets” OR “Fasting-Mimicking” OR “Fasting mimicking Diet” OR “Fasting mimicking Diets” OR “fasting mimicking.” The publications was exported in TXT format with “Full Record and Cited References.”

### 2.2 Bibliometric analysis and visualization

Once the data was compiled, the WoSCC Online Analysis Platform facilitated a detailed bibliometric analysis. This analysis centered around annual publication numbers, the top 10 in terms of productive countries/regions, authors, organizations, and journals, and spotlighted the top 20 high-citation publications. For a more visual representation and understanding of the data, VOSviewer 1.6.16 software was employed. The software enabled the mapping and visualization of several facets, such as co-authorships across institutions, countries/regions, and authors; citation patterns across journals and references; and co-occurrence patterns of keywords. The resultant visualizations, presented in the form of detailed maps and figures, were subsequently exported for further analysis and interpretation. In the analysis of keyword co-occurrence, we consolidated the synonyms “fasting mimicking diet” and “mimicking diet” under the single term “fasting-mimicking diet” for uniformity and clarity.

## 3 Results

### 3.1 Trends in global publications

**Figure 1:**
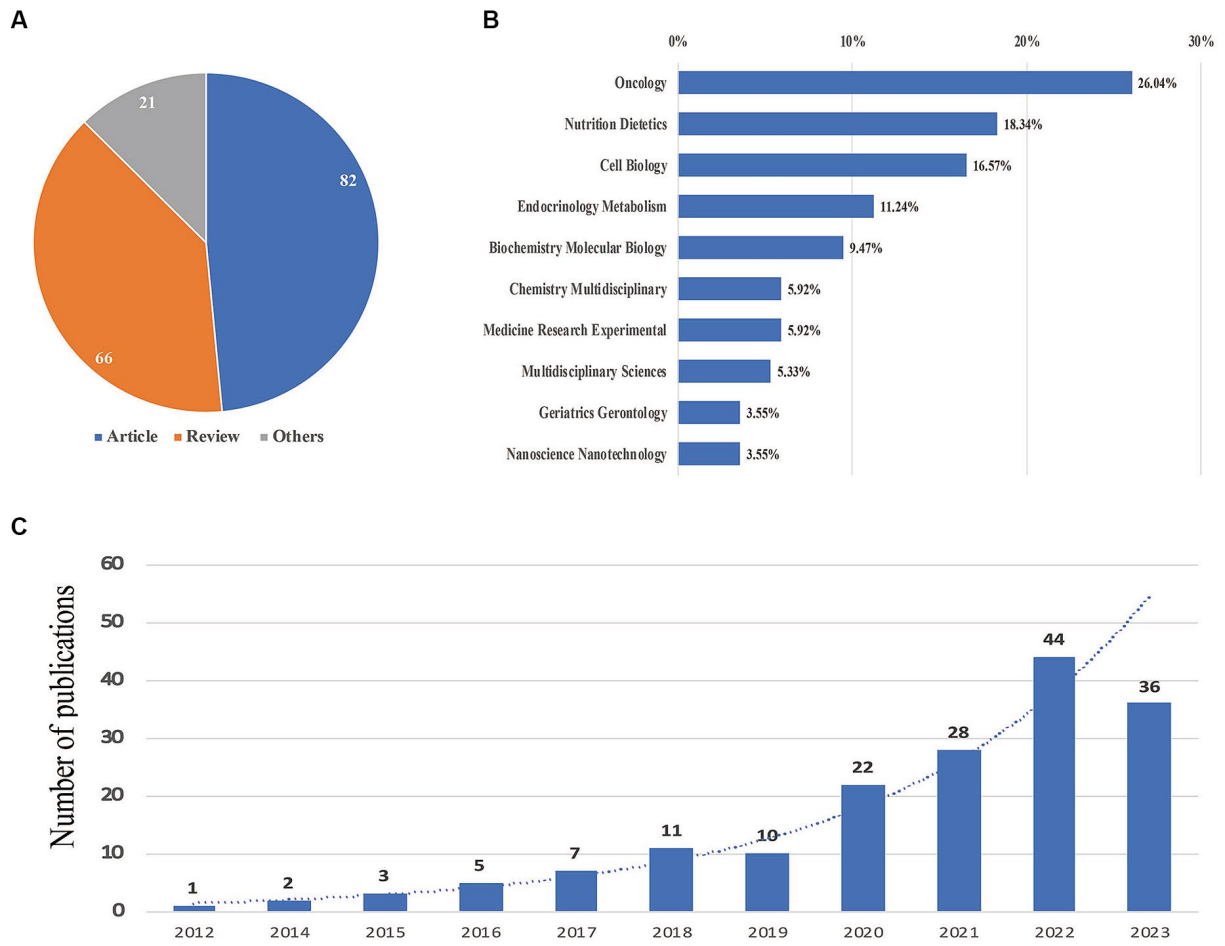
Yearly quantity and literature type of publications. **(A)** Literature type distribution. **(B)** Subject category distribution. **(C)** Annual publication quantitative distribution.

Based on the provided data related to the FMD, a total of 169 documents were identified. Figure 1 illustrates the trend in publications, the variety of publication types, and the categories of subjects being explored. Analyzing the time frames, the literature can be grouped into two primary phases: in the initial phase, which spans from 2012 to 2016, there was a modest number of publications, never surpassing 10 annually. This phase reflects the emerging interest and early research conducted on FMD. The subsequent phase from 2017 to 2023 represents a significant uptick in research intensity and focus. During this period, the yearly number of documents consistently increased, peaking at 44 publications in 2022. This increasing trend suggests a growing recognition of the relevance and significance of FMD in the scientific community. Projecting from current patterns, it is anticipated that publications related to FMD will continue on an upward trajectory due to the heightened interest and perceived importance of the topic. Diving into the specifics of publication types, the pie chart in Figure 1A reveals that out of the 169 publications, 82 were articles (representing 48.5% of the total), 66 were reviews (accounting for 39.1%), and the remaining 21 (or 12.4%) fell into other categories. As for the subject areas covered by these documents, Figure 1B presents a detailed breakdown: Oncology leads the chart with 26.64% publications, followed closely by Nutrition Dietetics at 18.34% and Cell Biology at 16.57%. The bar chart in Figure 1C offers a clear visualization of the yearly growth in FMD-related publications, emphasizing the exponential rise in research output from 2017 onwards.

### 3.2 Evaluation of countries/regions, academic organizations, authors, and journals

**Figure 2:**
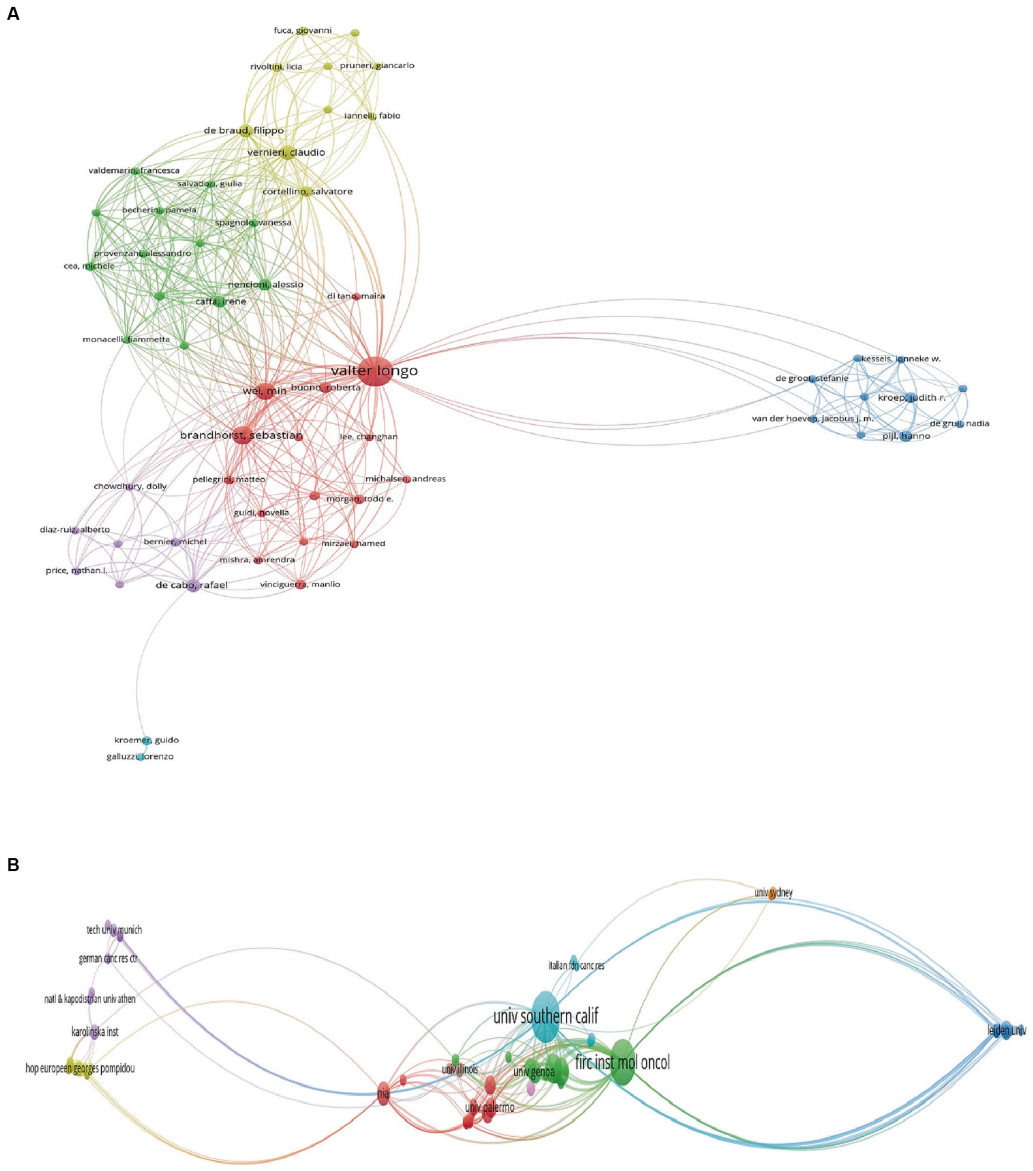
Visualization knowledge maps of authors and institutions. **(A)** The co-authorship map of authors. **(B)** The co-authorship map of institutions.

**Figure 3:**
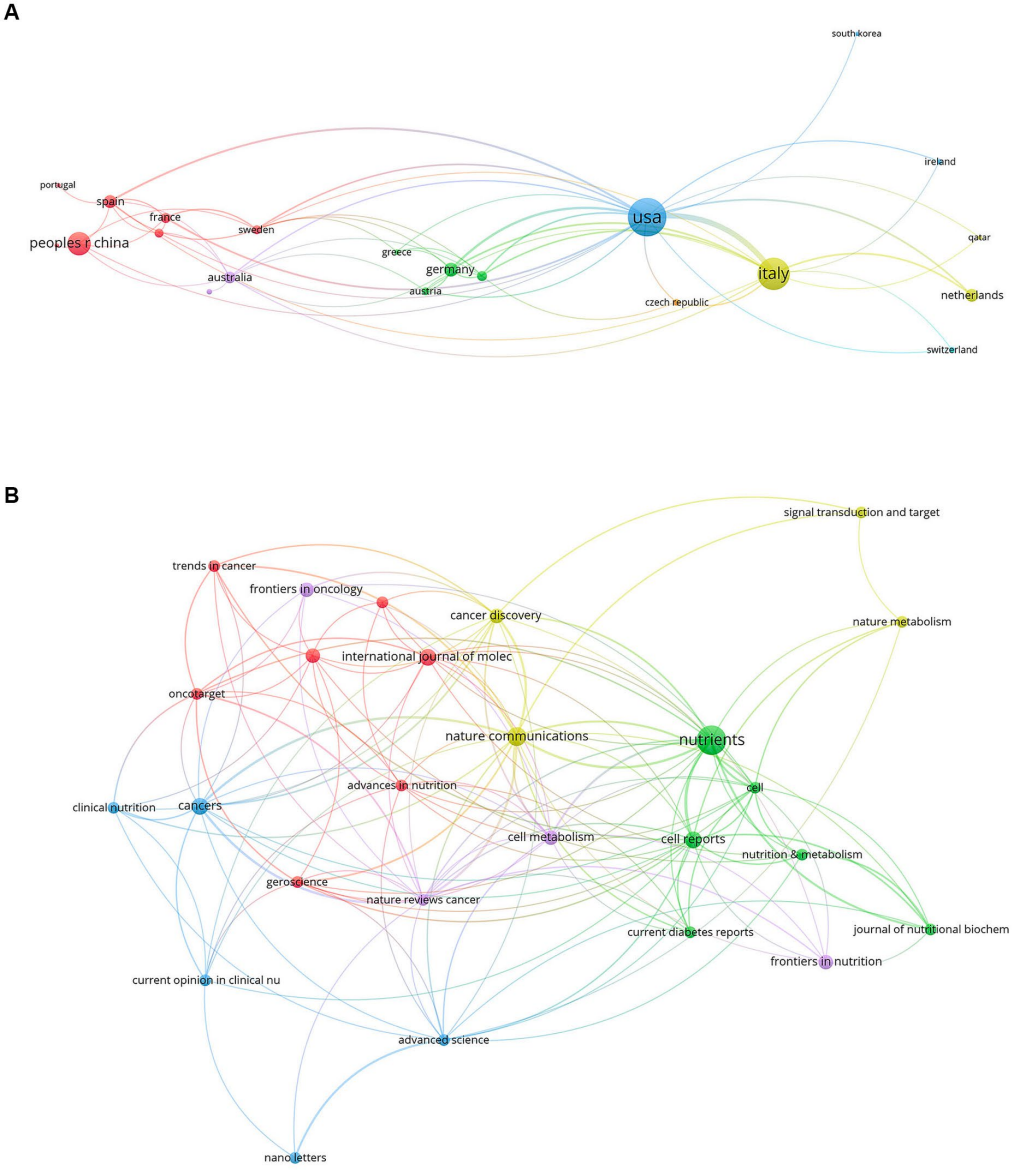
The visualization knowledge map of countries/regions and journals. **(A)** The co-authorship map of countries/regions. **(B)** The co-authorship map of journals.

In total, 945 authors from 342 institutions and 25 countries/regions contributed to the field of FMD in 111 journals. The most productive country was USA with 86 publications, followed by Italy (61 publications) and the People’s Republic of China (33 publications). For institutions, the University of Southern California tops the list with 45 publications, followed by the Ifom Fire Institute of Molecular Oncology with 42 publications, and the Chinese Academy of Sciences with 15 publications. In terms of individual researchers, Valter D. Longo stands out with 47 publications, showcasing him as a leading expert in this area. The concept of FMD was developed and popularized by he and his colleagues at the University of Southern California. Sebastian Brandhorst and Filippo De Braud follow with 17 and 14 publications, respectively. The most productive journal is Nutrients with 12 publications, and followed by the Nature Communications with 5 publications and cancers with 4 publications. Overall, this analysis underscores the importance and growing interest in the FMD in the global scientific community. Tables 1, 2 compile the 10 most prolific authors, organizations, countries/regions and journals in the FMD research arena. Figures 2, 3 present network visualization maps depicting citation interconnections among journals, countries/regions, institutions, and authors.

**Table 1:** The top 10 productive authors, institutions and countries based on publications.

Items	Publications				
	Ranking	Country	Number	Citations	H-index
Country	1	United States	86	4,619	30
	2	Italy	61	3,704	25
	3	Peoples R China	33	343	10
	4	Germany	12	1,066	5
	5	Netherlands	10	357	4
	6	Spain	10	177	7
	7	Australia	8	686	6
	8	England	6	961	4
	9	France	6	108	5
	10	Belgium	5	60	4
Institution	1	University of Southern California	49	3,976	24
	2	Ifom Firc Institute of Molecular Oncology	42	3,321	22
	3	Chinese Academy of Sciences	15	144	6
	4	Fondazione Irccs Istituto Nazionale Tumori Milan	14	391	8
	5	University of Chinese Academy of Sciences	13	126	5
	6	University of California System	12	763	6
	7	University of Milan	12	391	8
	8	National Institutes of Health NIH USA	11	1,201	8
	9	University of Genoa	11	617	8
	10	National Center for Nanoscience Technology China	9	33	4
Author	1	Valter D. Longo	41	3,871	23
	2	Sebastian Brandhorst	17	1,403	11
	3	Filippo De Braud	12	330	6
	4	Claudio Vernieri	11	384	8
	5	Valter Longo	8	29	3
	6	Alessio Nencioni	7	516	6
	7	Irene Caffa	7	516	6
	8	Wen Su	6	32	4
	9	Wenping Huang	6	18	2
	10	Roberta Buono	6	564	4

**Table 2:** The top 10 productive journals.

Ranking	Journal name	Country	Counts
1	Nutrients	Switzerland	12
2	Nature Communications	England	5
3	Cancers	Switzerland	4
4	Cell Reports	United States	4
5	International Journal of Molecular Sciences	Switzerland	4
6	Annals of Oncology	Netherlands	3
7	Cancer Discovery	United States	3
8	Cancer Research	United States	3
9	Cell Metabolism	United States	3
10	Frontiers in Nutrition	Switzerland	3
11	Frontiers in Oncology	Switzerland	3
12	Journal of Clinical Oncology	United States	3
13	Trends In Endocrinology and Metabolism	United States	3

### 3.3 Analysis of highly-cited publications

**Figure 4:**
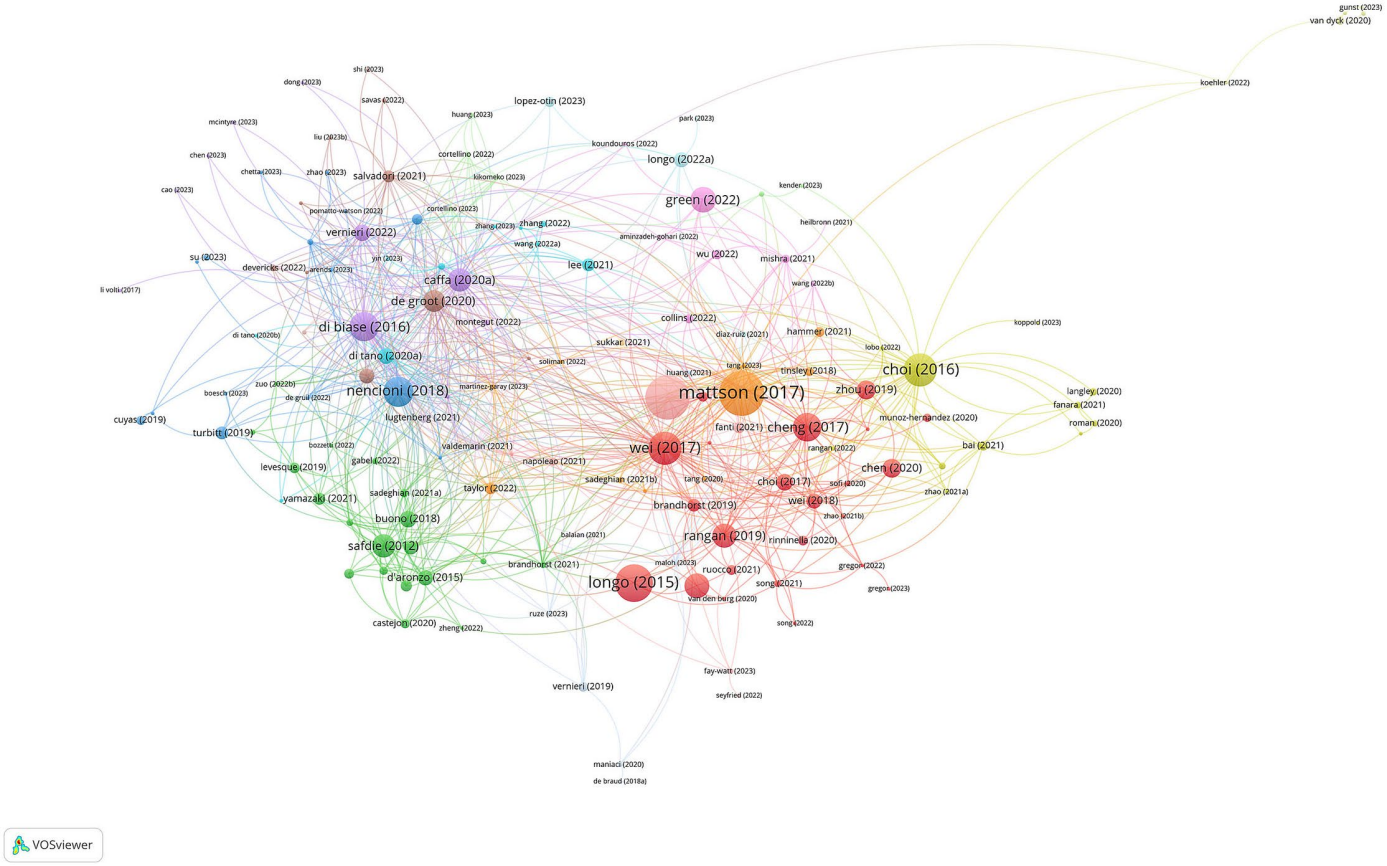
The visualized citations of references.

The visualized citations of references are depicted in Figure 4, and the attributes of the 20 most-cited documents are encapsulated in Table 3 (1, 2, 4, 6, 7, 10, 13, 15, 20–31). The first high-cited document was published in Ageing Research Reviews and authored by Mattson et al. (27). In the context of the FMD, the article may discuss how intermittent fasting, such as the FMD, can potentially promote various health benefits. These benefits may include improved metabolic health, weight management, and even potential effects on delaying aging processes and reducing the risk of chronic diseases. The second high-cited document was published in Cell Metabolism by Longo et al. (26). In this review, they explored the relationship between time-restricted feeding and fasting including the FMD and circadian rhythms, and their potential impacts on a healthy lifespan. These benefits might include improved metabolic health, cellular repair processes, and even longevity. The article likely delves into how aligning fasting periods with the body’s natural circadian rhythms and limiting food intake to specific time windows may positively impact health and longevity. The third high-cited document was published in Aging Cell by Longo et al. (25). The article may explore specific dietary intervention involving FMD, can influence aging processes, with the potential benefits of periodic fasting for promoting longevity and improving age-related health markers. The fourth high-cited document was published in Science Translational Medicine by Wei et al. (30). In this article, they investigate the effects of a FMD on various markers and risk factors associated with aging, diabetes, cancer, and cardiovascular disease, and explores how this diet can impact biomarkers related to these health conditions. It may discuss the potential benefits of the FMD in terms of improving these markers and reducing the risk factors associated with age-related diseases. The fifth high-cited document was published in Cell Reports by Choi et al. (13). They explore the effects of FMD on tissue regeneration, autoimmunity, and symptoms of multiple sclerosis (MS). They discuss the potential benefits of the FMD in promoting tissue regeneration and reducing autoimmunity, ultimately leading to a reduction in MS symptoms. This suggests that this specific dietary approach may hold promise in managing multiple sclerosis.

**Table 3:** The top 20 most highly cited references.

Rank	Title	Journal	Total citations	Year	First author
1	Impact of intermittent fasting on health and disease processes	Ageing Research Reviews	506	2017	Mark P. Mattson
2	Fasting, Circadian Rhythms, and Time-Restricted Feeding in Healthy Lifespan	Cell Metabolism	500	2016	Valter D. Longo
3	Interventions to Slow Aging in Humans: Are We Ready?	Aging Cell	359	2015	Valter D. Longo
4	Fasting-mimicking diet and markers/risk factors for aging, diabetes, cancer, and cardiovascular disease	Science Translational Medicine	276	2017	Min Wei
5	A Diet Mimicking Fasting Promotes Regeneration and Reduces Autoimmunity and Multiple Sclerosis Symptoms	Cell Reports	272	2016	In Young Choi
6	Fasting and cancer: molecular mechanisms and clinical application	Nature Reviews Cancer	233	2018	Alessio Nencioni
7	Fasting-Mimicking Diet Reduces HO-1 to Promote T Cell-Mediated Tumor Cytotoxicity	Cancer Cell	223	2016	Stefano Di Biase
8	Fasting-Mimicking Diet Promotes Ngn3-Driven β-Cell Regeneration to Reverse Diabetes	Cell	208	2017	Chia-Wei Cheng
9	Molecular mechanisms of dietary restriction promoting health and longevity	Nature Reviews Molecular Cell Biology	162	2022	Cara L. Green
10	Protein and amino acid restriction, aging and disease: from yeast to humans	Trends In Endocrinology And Metabolism	158	2014	Hamed Mirzaei
11	Fasting-Mimicking Diet Modulates Microbiota and Promotes Intestinal Regeneration to Reduce Inflammatory Bowel Disease Pathology	Cell Reports	144	2019	Priya Rangan
12	Fasting-mimicking diet and hormone therapy induce breast cancer regression	Nature	143	2020	Irene Caffa
13	Fasting enhances the response of glioma to chemo- and radiotherapy	Plos One	140	2012	Fernando Safdie
14	Fasting mimicking diet as an adjunct toneoadjuvant chemotherapy for breast cancer in the multicentre randomized phase 2 DIRECT trial	Nature Communications	124	2020	Stefanie de Groot
15	Neuroprotection of Fasting Mimicking Diet on MPTP-Induced Parkinson’s Disease Mice via Gut Microbiota and Metabolites	Neurotherapeutics	94	2019	Zhi-Lan Zhou
16	Gut Microbiota Metabolites in NAFLD Pathogenesis and Therapeutic Implications	International Journal of Molecular Sciences	91	2020	Jiezhong Chen
17	Fasting-Mimicking Diet Is Safe and Reshapes Metabolism and Antitumor Immunity in Patients with Cancer	Cancer Discovery	71	2022	Claudio Vernieri
18	Nutrition and fasting mimicking diets in the prevention and treatment of autoimmune diseases and immunosenescence	Molecular And Cellular Endocrinology	70	2017	In Young Choi
19	Starvation, Stress Resistance, and Cancer	Trends In Endocrinology And Metabolism	68	2018	Roberta Buono
20	Synergistic effect of fasting-mimicking diet and vitamin C against KRAS mutated cancers	Nature Communications	65	2020	Maira Di Tano

### 3.4 Analysis of keywords

**Figure 5:**
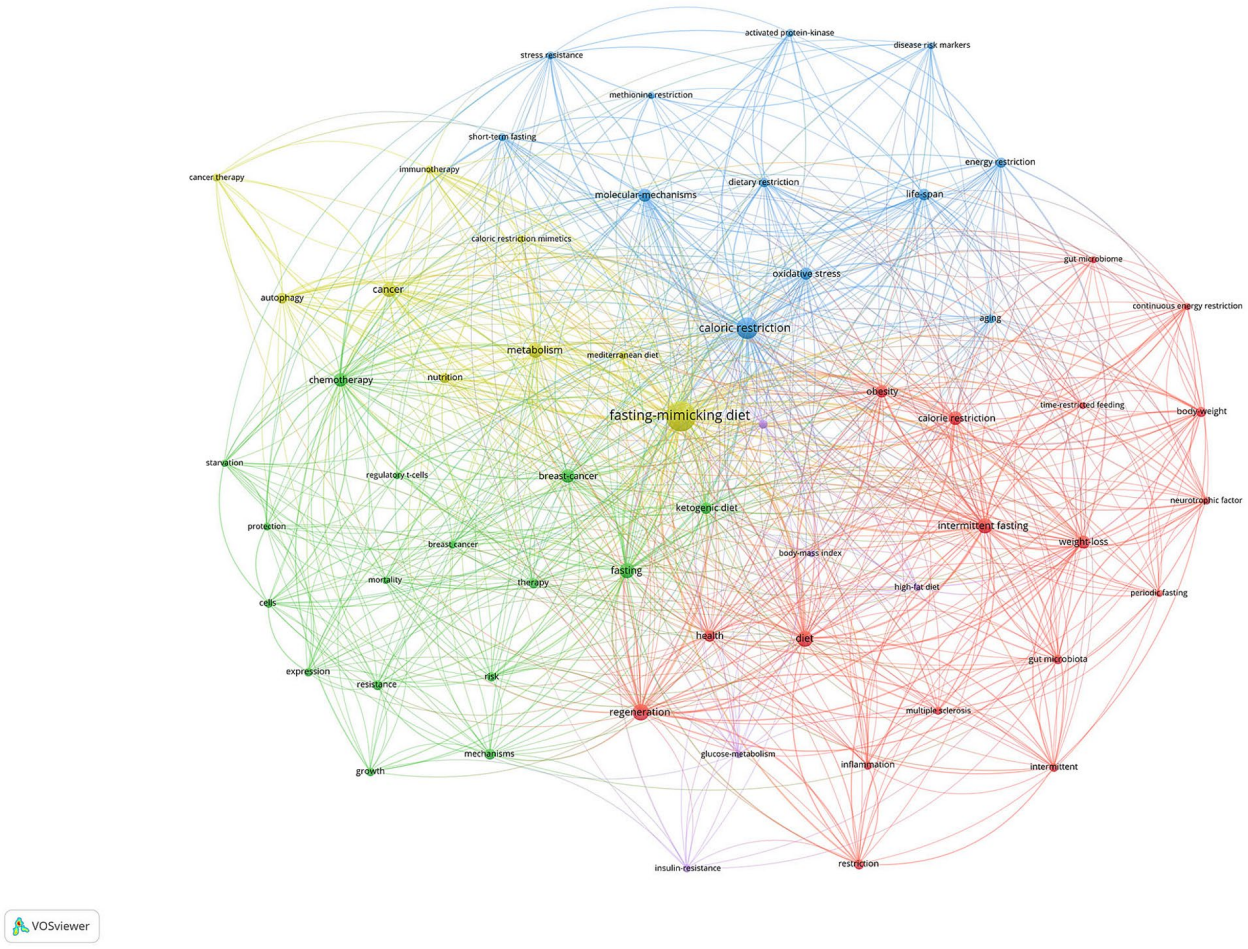
Visualization of keyword co-occurrence analysis.

Figure 5 displayed the network visualization maps of co-occurrence of keywords, including five clusters and key terms: Fasting and Diet Clusters: The central and prominent cluster revolves around “fasting-mimicking diet,” “caloric restriction,” “intermittent fasting,” and “ketogenic diet.” This is indicative of a core research area focused on dietary interventions and their impacts. Cancer and Treatment Cluster: On the left side, terms like “cancer,” “chemotherapy,” “cancer therapy,” and “breast cancer” are connected, suggesting research on how fasting or dietary interventions might affect cancer and its treatment. Metabolic Processes Cluster: Terms like “metabolism,” “glucose metabolism,” “insulin resistance,” and “oxidative stress” suggest a focus on the metabolic impacts of fasting and dietary restriction. Health Outcomes and Processes: “Regeneration,” “inflammation,” “resilience,” and “autophagy” point toward the potential health outcomes or cellular processes influenced by these diets. Other Dietary Patterns and Effects: There are terms like “body weight,” “weight loss,” and “energy restriction,” which relate to outcomes of dietary interventions and other dietary patterns.

## 4 Discussion

### 4.1 General information

To the best of our knowledge, this is the first bibliometric analysis related to the FMD. FMD is a specialized dietary regimen, has attracted growing attention in the scientific community due to its potential health benefits. Numerous studies have explored its implications for metabolic health, weight management, and potential in influencing the course of various diseases, including cancer and diabetes. In our study, there were a total of 169 publications related to FMD. From a geographical standpoint, the USA emerged as the leading contributor with 86 publications. The most productive institution and author are the University of Southern California and Valter D. Longo. The journal “Nutrients” was particularly noteworthy, with the highest number of FMD-related publications. Furthermore, the most highly cited work in this domain was published in Ageing Research Reviews by Mattson et al. (27), delving into the broader theme of intermittent fasting, encompassing FMD. In essence, the research on the FMD reflects a dynamic and growing field, with key players and institutions paving the way for future explorations and discoveries.

### 4.2 Hotspots and frontiers

On the basis of publications of the FMD, highly-cited publications, and important keywords with high frequency, the key research areas within the field of FMD have been outlined as follows.

#### 4.2.1 FMD for cancer

For this field, the FMD for cancer is the most popular topic. Many studies investigate the effects of FMD for cancer. For example, Caffa et al. (6) demonstrates the efficacy of combining a FMD (FMD) with hormone therapy to potentiate cancer treatment in hormone-receptor-positive breast cancers, which constitute roughly 75% of all breast cancer cases. In mouse models, the FMD synergistically improved the cancer-fighting impact of endocrine therapies, such as tamoxifen and fulvestrant, by significantly lowering systemic levels of IGF-1, insulin, and leptin, and impeding AKT–mTOR signaling pathways through the increased expression of EGR1 and PTEN. This combination, particularly when a cycle of FMD was added to a regimen of fulvestrant and palbociclib (a CDK4/6 inhibitor), not only induced durable tumor regression but also countered the development of acquired resistance to the medications. Additionally, it prevented tamoxifen-induced endometrial hyperplasia, a common side effect of this drug. Encouragingly, early-phase clinical observations in human patients mirrored the metabolic alterations seen in mice and were associated with prolonged anti-cancer benefits. These findings underline the potential of incorporating periodic FMDs as a complementary approach to enhance the effectiveness of hormone therapies in treating hormone-receptor-positive breast cancers (6).

Subsequently, the phase 2 DIRECT trial (7), which was the first randomized controlled study evaluating the effects of an FMD on toxicity and efficacy of chemotherapy in patients with cancer. In this study, they evaluated the effects of the FMD as an accompaniment to neoadjuvant chemotherapy in breast cancer patients. 131 HER2-negative stage II/III breast cancer patients participated, with half receiving an FMD and the other half their regular diet. Results showed no notable difference in toxicity levels between the two groups, even though one group did not receive dexamethasone with their treatment. The FMD group exhibited a higher likelihood of a significant or complete radiological response to chemotherapy. Importantly, DNA damage in T-lymphocytes was also reduced in the FMD group, hinting at the diet’s protective qualities. Conclusively, the FMD displayed potential in enhancing the effects of chemotherapy in early-stage breast cancer patients, making it worthy of further exploration in cancer treatment protocols (7).

In addition, FMD may be also benefits for other types of cancer besides breast cancer, including lung cancer (32), colorectal cancer (3), and prostate cancer (33). There’s been some benefits in the FMD for cancer: Protective effect during chemotherapy: Preclinical studies suggest that cycles of fasting or FMD can render cancer cells more sensitive to chemotherapy while protecting healthy cells. This is termed differential stress resistance (DSR). By enhancing the effects of chemotherapy on cancer cells and reducing side effects on normal cells, the quality of life for patients undergoing chemotherapy might be improved. Cancer cell vulnerability: Some preclinical studies have indicated that fasting or FMD might slow down the growth of certain types of tumors. This is believed to be because cancer cells, which are primed for rapid growth and are more metabolically active, become more vulnerable under nutrient-scarce conditions created by fasting or FMD. Reduced inflammation: Prolonged fasting and FMD can reduce levels of circulating IGF-1 (insulin-like growth factor-1), a hormone linked to cancer risk, and promote a switch to a state that reduces inflammation, which can potentially decrease the chances of cancer development and progression. Stem cell regeneration: Fasting or FMD has been shown in mouse models to stimulate the regeneration of new stem cells in various systems, including the hematopoietic system. This could be beneficial for recovery after chemotherapy, which often damages the bone marrow and immune cells (4, 34–42).

#### 4.2.2 FMD for metabolic-related diseases

Besides for cancer, FMD may has some potential benefits for patients with metabolic-related diseases such as diabetes and NAFLD. Cheng et al. (4) found a 4-day fasting mimicking diet (FMD) in mice has been shown to stimulate the regeneration of insulin-producing β cells, akin to processes seen during pancreatic development. This FMD regimen can rejuvenate insulin secretion and balance glucose levels in both type 1 and type 2 diabetes mouse models. In human pancreatic islets with type 1 diabetes, fasting conditions decrease PKA and mTOR activity, leading to the expression of specific genes and subsequent insulin production. However, these FMD effects can be countered by IGF-1, but similarly induced by inhibiting PKA and mTOR. Essentially, the study suggests that an FMD can reprogram pancreatic cells to renew insulin production in type 1 diabetes patients’ islets and mitigate symptoms of both type 1 and type 2 diabetes in mice (4).

The potential Benefits of FMD for Metabolic Diseases including, weight Loss: FMD has been shown to help in reducing body weight. This can be beneficial for metabolic conditions where weight plays a significant role, such as Type 2 diabetes and metabolic syndrome. Blood Sugar Control: Some studies have suggested that FMD can help regulate blood sugar levels, making it potentially beneficial for those with insulin resistance or Type 2 diabetes. Cardiovascular Health: FMD might improve lipid profiles by reducing levels of bad cholesterol and increasing good cholesterol, which is beneficial for heart health. Cellular Autophagy: Fasting or diets mimicking fasting can promote autophagy, the body’s natural process of cleaning out damaged cells, which might offer protective benefits against several metabolic-related diseases (5, 9, 30, 43, 44).

#### 4.2.3 FMD for cognitive improvement

FMD has the potential effect on the improvement of cognitive functions in Alzheimer’s disease (AD). Rangan et al. (16) investigated the effects of FMD on cognitive functions in Alzheimer’s models, and found that FMD cycles demonstrated a reduction in cognitive decline and AD pathology in two mouse models, outperforming the effects of protein restriction cycles. In one particular mouse model, the FMD significantly reduced signs of AD, such as brain plaques and tau tangles, and fostered the growth of neural stem cells (16).

Moreover, FMD decreased the number of microglia and reduced the expression of neuroinflammatory genes, including the superoxide-producing Nox2. Improved cognition was observed in mice lacking Nox2 or those treated with a Nox2 inhibitor. Preliminary clinical data also suggest that FMD cycles are feasible and generally safe for a subset of AD patients. These findings underscore the potential of FMD cycles in delaying cognitive decline in AD (16, 45, 46).

There were some limitations in our study. We exclusively utilized the WoSCC database, as the VOSviewer software is incompatible with other databases like Embase and Pubmed for analyzing and visualizing co-citation maps. Furthermore, the volume of publications concerning FMD is still quite limited, and the pre-clinical and clinical data on both sexes and to elucidate potential differences are lacked, necessitating the imperative for additional research. Expanding this body of work is crucial to enhance our understanding of FMD’s impacts on human health and to optimize the use of this method for health improvement purposes.

In conclusion, this is the first bibliometric analysis of the FMD. The main research hotspots and frontiers were FMD for cancer, FMD for metabolic-related diseases, and FMD for cognitive improvement. FMD may have some potential benefits for multiple diseases, including cancer, diabetes, and Alzheimer’s diseases, which should be further investigated.

## Data availability statement

The raw data supporting the conclusions of this article will be made available by the authors, without undue reservation.

## Author contributions

XL: Data curation, Formal Analysis, Methodology, Project administration, Supervision, Validation, Writing – original draft, Writing – review & editing. YG: Formal Analysis, Funding acquisition, Project administration, Resources, Validation, Visualization, Writing – original draft, Writing – review & editing.

## References

[1] di Biase S Lee C Brandhorst S Manes B Buono R Cheng CW. Fasting-mimicking diet reduces HO-1 to promote T cell-mediated tumor cytotoxicity. Cancer Cell. (2016) 30:136–46. doi: 10.1016/j.ccell.2016.06.005, PMID: 27411588 PMC5388544

[2] Vernieri C Fucà G Ligorio F Huber V Vingiani A Iannelli F. Fasting-mimicking diet is safe and reshapes metabolism and antitumor immunity in patients with Cancer. Cancer Discov. (2022) 12:90–107. doi: 10.1158/2159-8290.CD-21-0030, PMID: 34789537 PMC9762338

[3] Zhong Z Zhang H Nan K Zhong J Wu Q Lu L. Fasting-mimicking diet drives antitumor immunity against colorectal cancer by reducing IgA-producing cells. Cancer Res. (2023) 83:3529–43. doi: 10.1158/0008-5472.CAN-23-0323, PMID: 37602826 PMC10618736

[4] Cheng CW Villani V Buono R Wei M Kumar S Yilmaz OH. Fasting-mimicking diet promotes Ngn3-driven β-cell regeneration to reverse diabetes. Cells. (2017) 168:775–788.e12. doi: 10.1016/j.cell.2017.01.040, PMID: 28235195 PMC5357144

[5] Hammer SS Vieira CP McFarland D Sandler M Levitsky Y Dorweiler TF. Fasting and fasting-mimicking treatment activate SIRT1/LXRα and alleviate diabetes-induced systemic and microvascular dysfunction. Diabetologia. (2021) 64:1674–89. doi: 10.1007/s00125-021-05431-5, PMID: 33770194 PMC8236268

[6] Caffa I Spagnolo V Vernieri C Valdemarin F Becherini P Wei M. Fasting-mimicking diet and hormone therapy induce breast cancer regression. Nature. (2020) 583:620–4. doi: 10.1038/s41586-020-2502-7, PMID: 32669709 PMC7881940

[7] de Groot S Lugtenberg RT Cohen D Welters MJP Ehsan I Vreeswijk MPG. Fasting mimicking diet as an adjunct toneoadjuvant chemotherapy for breast cancer in the multicentre randomized phase 2 DIRECT trial. Nat Commun. (2020) 11:3083. doi: 10.1038/s41467-020-16138-3, PMID: 32576828 PMC7311547

[8] Vernieri C Ligorio F Zattarin E Rivoltini L de Braud F. Fasting-mimicking diet plus chemotherapy in breast cancer treatment. Nat Commun. (2020) 11:4. doi: 10.1038/s41467-020-18194-1 32848145 PMC7450058

[9] Mishra A Mirzaei H Guidi N Vinciguerra M Mouton A Linardic M. Fasting-mimicking diet prevents high-fat diet effect on cardiometabolic risk and lifespan. Nat Metab. (2021) 3:1342. doi: 10.1038/s42255-021-00469-6, PMID: 34650272

[10] Nencioni A Caffa I Cortellino S Longo VD. Fasting and cancer: molecular mechanisms and clinical application. Nat Rev Cancer. (2018) 18:707–19. doi: 10.1038/s41568-018-0061-0, PMID: 30327499 PMC6938162

[11] Huang AW Wei M Caputo S Wilson ML Antoun J Hsu WC. An intermittent fasting mimicking nutrition Bar extends physiologic ketosis in time restricted eating: a randomized, controlled, parallel-arm study. Nutrients. (2021) 13:11. doi: 10.3390/nu13051523 PMC8147148 33946428

[12] Videja M Sevostjanovs E Upmale-Engela S Liepinsh E Konrade I Dambrova M. Fasting-mimicking diet reduces trimethylamine N-oxide levels and improves serum biochemical parameters in healthy volunteers. Nutrients. (2022) 14:13. doi: 10.3390/nu14051093 PMC8912301 35268068

[13] Choi IY Piccio L Childress P Bollman B Ghosh A Brandhorst S. A diet mimicking fasting promotes regeneration and reduces autoimmunity and multiple sclerosis symptoms. Cell Rep. (2016) 15:2136–46. doi: 10.1016/j.celrep.2016.05.009, PMID: 27239035 PMC4899145

[14] Cortellino S Raveane A Chiodoni C Delfanti G Pisati F Spagnolo V. Fasting renders immunotherapy effective against low-immunogenic breast cancer while reducing side effects. Cell Rep. (2022) 40:111256. doi: 10.1016/j.celrep.2022.111256 36001966

[15] Rangan P Choi I Wei M Navarrete G Guen E Brandhorst S. Fasting-mimicking diet modulates microbiota and promotes intestinal regeneration to reduce inflammatory bowel disease pathology. Cell Rep. (2019) 26:2704. doi: 10.1016/j.celrep.2019.02.019, PMID: 30840892 PMC6528490

[16] Rangan P Lobo F Parrella E Rochette N Morselli M Stephen TL. Fasting-mimicking diet cycles reduce neuroinflammation to attenuate cognitive decline in Alzheimer’s models. Cell Rep. (2022) 40:111417. doi: 10.1016/j.celrep.2022.111417 36170815 PMC9648488

[17] Lin X Wang S Huang J. A bibliometric analysis of alternate-day fasting from 2000 to 2023. Nutrients. (2023) 15:724. doi: 10.3390/nu15173724, PMID: 37686756 PMC10490218

[18] Kiss A Temesi Á Tompa O Lakner Z Soós S. Structure and trends of international sport nutrition research between 2000 and 2018: bibliometric mapping of sport nutrition science. J Int Soc Sports Nutr. (2021) 18:12. doi: 10.1186/s12970-021-00409-5, PMID: 33546728 PMC7866438

[19] Zyoud SH Shakhshir M Abushanab AS al-Jabi SW Koni A Shahwan M. Mapping the global research landscape on nutrition and the gut microbiota: visualization and bibliometric analysis. World J Gastroenterol. (2022) 28:2981–93. doi: 10.3748/wjg.v28.i25.2981, PMID: 35978868 PMC9280741

[20] Buono R Longo VD. Starvation, stress resistance, and Cancer. Trends Endocrinol Metab. (2018) 29:271–80. doi: 10.1016/j.tem.2018.01.008, PMID: 29463451 PMC7477630

[21] Chen JZ Vitetta L. Gut microbiota metabolites in NAFLD pathogenesis and therapeutic implications. Int J Mol Sci. (2020) 21:5214. doi: 10.3390/ijms21155214 32717871 PMC7432372

[22] Choi IY Lee C Longo VD. Nutrition and fasting mimicking diets in the prevention and treatment of autoimmune diseases and immunosenescence. Mol Cell Endocrinol. (2017) 455:4–12. doi: 10.1016/j.mce.2017.01.042, PMID: 28137612 PMC5862044

[23] di Tano M Raucci F Vernieri C Caffa I Buono R Fanti M. Synergistic effect of fasting-mimicking diet and vitamin C against <i>KRAS</i> mutated cancers. Nat Commun. (2020) 11:3. doi: 10.1038/s41467-020-16243-3, PMID: 32393788 PMC7214421

[24] Green CL Lamming DW Fontana L. Molecular mechanisms of dietary restriction promoting health and longevity. Nat Rev Mol Cell Biol. (2022) 23:56–73. doi: 10.1038/s41580-021-00411-4, PMID: 34518687 PMC8692439

[25] Longo VD Antebi A Bartke A Barzilai N Brown-Borg HM Caruso C. Interventions to slow aging in humans: are we ready? Aging Cell. (2015) 14:497–510. doi: 10.1111/acel.12338, PMID: 25902704 PMC4531065

[26] Longo VD Panda S. Fasting, circadian rhythms, and time-restricted feeding in healthy lifespan. Cell Metab. (2016) 23:1048–59. doi: 10.1016/j.cmet.2016.06.001, PMID: 27304506 PMC5388543

[27] Mattson MP Longo VD Harvie M. Impact of intermittent fasting on health and disease processes. Ageing Res Rev. (2017) 39:46–58. doi: 10.1016/j.arr.2016.10.005, PMID: 27810402 PMC5411330

[28] Mirzaei H Suarez JA Longo VD. Protein and amino acid restriction, aging and disease: from yeast to humans. Trends Endocrinol Metab. (2014) 25:558–66. doi: 10.1016/j.tem.2014.07.002, PMID: 25153840 PMC4254277

[29] Safdie F Brandhorst S Wei M Wang W Lee C Hwang S. Fasting enhances the response of glioma to chemo- and radiotherapy. PLoS One. (2012) 7:9. doi: 10.1371/journal.pone.0044603 PMC3439413 22984531

[30] Wei M Brandhorst S Shelehchi M Mirzaei H Cheng CW Budniak J. Fasting-mimicking diet and markers/risk factors for aging, diabetes, cancer, and cardiovascular disease. Sci Transl Med. (2017) 9:12. doi: 10.1126/scitranslmed.aai8700 PMC6816332 28202779

[31] Zhou ZL Jia XB Sun MF Zhu YL Qiao CM Zhang BP. Neuroprotection of fasting mimicking diet on MPTP-induced Parkinson’s disease mice via gut microbiota and metabolites. Neurotherapeutics. (2019) 16:741–60. doi: 10.1007/s13311-019-00719-2, PMID: 30815845 PMC6694382

[32] Cortellino S Quagliariello V Delfanti G Blaževitš O Chiodoni C Maurea N. Fasting mimicking diet in mice delays cancer growth and reduces immunotherapy-associated cardiovascular and systemic side effects. Nat Commun. (2023) 14:5529. doi: 10.1038/s41467-023-41066-3, PMID: 37684243 PMC10491752

[33] Fay-Watt V O’Connor S Roshan D Romeo AC Longo VD Sullivan FJ. Correction: the impact of a fasting mimicking diet on the metabolic health of a prospective cohort of patients with prostate cancer: a pilot implementation study. Prostate Cancer Prostatic Dis. (2023) 26:435. doi: 10.1038/s41391-022-00540-7, PMID: 35585259 PMC10247363

[34] McIntyre CL Temesgen A Lynch L. Diet, nutrient supply, and tumor immune responses. Trends Cancer. (2023) 9:752–63. doi: 10.1016/j.trecan.2023.06.003, PMID: 37400315

[35] Martínez-Garay C Djouder N. Dietary interventions and precision nutrition in cancer therapy. Trends Mol Med. (2023) 29:489–511. doi: 10.1016/j.molmed.2023.04.004, PMID: 37263858

[36] Liu XX Peng SY Tang GN Xu G Xie Y Shen D. Fasting-mimicking diet synergizes with ferroptosis against quiescent, chemotherapy-resistant cells. EBioMedicine. (2023) 90:104496. doi: 10.1016/j.ebiom.2023.104496 36863257 PMC9996234

[37] de Gruil N Pijl H van der Burg SH Kroep JR. Short-term fasting synergizes with solid Cancer therapy by boosting antitumor immunity. Cancer. (2022) 14:16. doi: 10.3390/cancers14061390 PMC8946179 35326541

[38] Su W Tan M Wang Z Zhang J Huang W Song H. Targeted degradation of PD-L1 and activation of the STING pathway by carbon-dot-based PROTACs for Cancer immunotherapy. Angew Chem Int Ed Engl. (2023) 62:e202218128. doi: 10.1002/anie.202218128, PMID: 36647763

[39] Ligorio F Provenzano L Vernieri C. Fasting-mimicking diet: a metabolic approach for the treatment of breast cancer. Curr Opin Oncol. (2023) 35:491–9. doi: 10.1097/CCO.0000000000000986, PMID: 37621169

[40] Li T Yue Y Ma Y Zhong Z Guo M Zhang J. Fasting-mimicking diet alleviates inflammatory pain by inhibiting neutrophil extracellular traps formation and neuroinflammation in the spinal cord. Cell Commun Signal. (2023) 21:250. doi: 10.1186/s12964-023-01258-2, PMID: 37735678 PMC10512659

[41] Blaževitš O Di Tano M Longo VD. Fasting and fasting mimicking diets in cancer prevention and therapy. Trends Cancer. (2023) 9:212–22. doi: 10.1016/j.trecan.2022.12.006, PMID: 36646607

[42] Arends J. Caloric restriction and fasting-mimicking diets in the treatment of cancer patients. Curr Opin Clin Nutr Metab Care. (2023) 26:423–9. doi: 10.1097/MCO.0000000000000959, PMID: 37389467

[43] Zhao N Gao YF Bao L Lei J An HX Pu FX. Glycemic control by umbilical cord-derived mesenchymal stem cells promotes effects of fasting-mimicking diet on type 2 diabetic mice. Stem Cell Res Ther. (2021) 12:395. doi: 10.1186/s13287-021-02467-7, PMID: 34256832 PMC8278637

[44] Wei S Han R Zhao J Wang S Huang M Wang Y. Intermittent administration of a fasting-mimicking diet intervenes in diabetes progression, restores β cells and reconstructs gut microbiota in mice. Nutr Metab. (2018) 15:80. doi: 10.1186/s12986-018-0318-3, PMID: 30479647 PMC6245873

[45] Boccardi V Pigliautile M Guazzarini AG Mecocci P. The potential of fasting-mimicking diet as a preventive and curative strategy for Alzheimer’s disease. Biomol Ther. (2023) 13:1133. doi: 10.3390/biom13071133, PMID: 37509169 PMC10377404

[46] Lobo F Haase J Brandhorst S. The effects of dietary interventions on brain aging and neurological diseases. Nutrients. (2022) 14:5086. doi: 10.3390/nu14235086, PMID: 36501116 PMC9740746

